# Severity of gambling behaviors: exploring associations with venues, legality, and substance use

**DOI:** 10.1186/s13584-024-00604-0

**Published:** 2024-04-16

**Authors:** Hagit Bonny-Noach

**Affiliations:** 1 Head of Addictions Research Lab, Faculty of Social Sciences and Humanities, Department of Criminology, Ariel University, Ariel, Israel; 2 Board Member of the Israeli Society of Addiction Medicine (ILSAM), Ramat-Gan, Israel

**Keywords:** Gambling, Gambling policy, Problem gambling, Legalize, Substance use

## Abstract

**Introduction:**

The COVID-19 pandemic has been linked to an increase in gambling behaviors, potentially leading to Gambling Disorder (GD) and adverse health and social consequences. Problematic gambling has received little research attention over the years in Israeli society and the issue of gambling is not high on the list of priorities of Israeli policymakers. The present study examined gambling behavior in Israel on a continuum of severity and its association with venues where the gambling occurs, legality, attitude toward the legalization of casinos and poker, and substance use.

**Methods:**

The study questionnaires were distributed to approximately 15,000 Jewish-Israeli adults via internet panel. From 3,088 Israeli adults who answered the questionnaire, 1,251 (40.5%) reported gambling in the last year and were included in the analyses.

**Results:**

Based on the Problem Gambling Severity Index, 60% of participants were classified as non-problem gamblers, 25% as at low-risk for a gambling problem, 11% as at moderate risk, and 4% as having a gambling problem. Most online gambling was practiced by non-problem gamblers (40%) and most illegal gambling was by low-risk gamblers (34%). The more severe the gambling behavior was on the continuum, the more it was associated with illegal substance use and positive attitudes toward legalized casinos in Israel. Logistic regression showed the odds of developing moderate and problem gambling were 3.8 times higher for online gamblers (OR = 3.8; CI 2.6–5.4; *p* < 0.000) and 3.3 times higher for illegal gamblers (OR = 3.3; CI 2.2–4.9; *p* < 0.000).

**Conclusion:**

Though more research attention should be paid to gambling behaviors, harm reduction gambling interventions should be made available to all categories on the continuum of severity of gambling behaviors. The present study provides evidence-based information to promote health policies that aim to prevent and reduce harm for Israeli gamblers.

## Introduction

Many adults participate in gambling as a social activity and for fun without any adverse consequences [1, 2]. However, this can sometimes lead to problem gambling behavior and cause significant personal and social harm [3], such as significant distress, physical and mental health problems, and financial, relationship, and legal problems [4].

In 2013, gambling disorder (GD) was placed in the 5th DSM [5] along with substance use disorders in the category of ‘Substance-related and addictive disorders’, and gambling disorder has recently been included in the category of ‘Disorders due to substance use or addictive behaviors’ in ICD-11 [6]. Despite this, unlike the systematic global data monitoring conducted regarding substances such as drugs, collected in the United Nations Office of Drugs and Crime (UNODC) [7], and alcohol, monitored by the World Health Organization (WHO) [8], there is no global organization that collects and monitors consistent global data regarding gambling [9]. As every country has a unique gambling market that is affected by the national legislative policy and political and social local cultural climate [10], each country has a different rate of people who identify with ‘problem gambling’. Data regarding the extent of people with ‘problem gambling’ were derived from studies conducted in different countries, and the global estimates for ‘problem gambling’ range from 0.12–5.8% worldwide [1]. These estimates of problem gambling have been accepted by the WHO, which publishes epidemiology estimated data in 2017, pointing to 350 million people with gambling problems worldwide every year [11].

In Israel gambling has received little research attention over the years. The issue of gambling is not high on the list of priorities of Israeli policymakers, and there is no comprehensive policy associated with gambling [9, 13].

Israel is a secular Jewish democratic state, although there is no legal or political separation between religion and state. Therefore, it follows a conservative policy toward gambling because the traditional religious parties oppose it [14]. Israeli law permits certain types of gambling through two operators: the National Lottery (‘Mifal Hapais’), which offers various physical (Offline gambling takes place in land-based settings) daily or weekly sweepstakes (include the Israel Lottery, which offers various types of lotto games and scratch cards), and the Israel Sports Betting Board (ISBB) (‘Winner-Toto’), which runs on-site and online sports betting. Although there is no online national lottery, it is possible to become a member of a ‘club lottery group’ that collects gambling money from their member gamblers online and buys for them a legal lottery ticket from the official operator in Israel. The official national lottery operator does not endorse this semi-illegal gambling method and has attempted to restrict it, albeit with limited success. All other forms of gambling which are not under the aegis of the National Lottery or ISBB are considered illegal. Several of these are flourishing in Israel, including ‘Bettim’ (illegal sports betting), poker, animal betting, and dice [13].

Israel is considered a conservative country, especially in the context of establishing a casino [15]. There have been unsuccessful attempts to establish a casino in Israel, mostly during the 1990s; and several bills to allow poker tournaments in Israel in recent years have not been successful [9]. Since 2014, the National Lottery has been conducting surveys on gambling behaviors within a representative sample of the Israeli population. According to the latest survey conducted in 2022, the estimated prevalence of ‘problem gambling’ among the entire Israeli population stands at 2.5%. Notably, this figure increases to 7% when considering only those who reported engaging in gambling activities within the past year [16]. However, these surveys are considered grey literature and are accessible on the National Lottery website and have not undergone peer-reviewed journal publication to date [17]. In another recent study among a representative sample of the Israeli population, 6.3% were categorized as moderate-risk and problem gamblers. Notably, among those who reported engaging in gambling within the past year, the percentage of moderate-risk and problem gamblers increased to 12.8% [17].

Despite the absence of a comprehensive policy related to gambling in Israeli society [9, 13], the concern for establishing treatment services for individuals dealing with gambling addiction has gained prominence in recent years. In 2019, an initiative led by the Ministry of Finance resulted in the formation of an inter-ministerial committee dedicated to addressing the treatment, enhancement, and development of the system for individuals struggling with gambling addiction [18]. Following the committee’s recommendations, and under the supervision of the Ministry of Welfare and Social Services and the Ministry of Health, there was an expansion of therapeutic options, accompanied by the establishment of new treatment centers for gamblers [19].

In the gambling literature, there has been a significant focus on studies of problem gambling behaviors and related harms [20, 21]. Additionally, policy and interventions have focused almost exclusively on problem gambling [22]. However, recent findings at a population level also identified that other categories on the continuum of severity, such as low and moderate risk gambling behaviors, can harm and affect others such as family members and friends [23, 24].

One potential accelerating factor for problematic gambling during recent years is the internet, which has changed the gambling environment since the beginning of the 21st century [25]. The internet offers different kinds of gambling and online applications that are faster, more attractive due to a variety of design and marketing options, less costly, and potentially more addictive than physical gambling [12]. Online easy access gambling platforms increase the opportunity to gamble and may contribute to rapid increase in problematic gambling and gambling disorder [26]. Problematic gambling and health risks related to gambling are higher among illegal gamblers than among legal gamblers [13, 27]. According to gender differences, the prevalence of gambling and gambling problems is higher among men than women [ 28, 29]. Additionally, in the field of addiction research, comorbidity of gambling disorder and substance use disorder is a well-documented phenomenon [30, 31]; and high levels of substance use have been found in people with gambling problems [32, 33]. This association was identified mostly in individuals with gambling disorder and problematic gambling. The association of non-severe gambling behaviors, such as low-to-moderate gambling, and substance use has been studied only to a limited extent.

As less attention is given to other gambling behavior categories on the continuum of severity and little is published according to gambling behavior in Israel, this study’s aims are as follows:

(1) Examine gambling behavior on the continuum of severity among gamblers in Israel; (2) Examine the association between gambling behavior on the continuum of severity and the venues used by the gambler (online or on-site); (3) Examine the association between gambling behavior on the continuum of severity with its legality (legal or illegal); (4) Examine the association between gambling behavior on the continuum of severity and gamblers’ attitudes toward legalization of casinos and poker in Israel; (5) Examine the association between gambling behavior on the continuum of severity and gamblers’ ever use of cannabis and other illegal substances; and (6) Characterize who is higher risk in gambling severity by socioeconomic characteristics, venues and legality.

## Methods

### Participants

The study questionnaires were distributed to approximately 15,000 Jewish-Israeli adults via internet panel. Of the 3,088 people who answered the questionnaire, 1,251 (40.5%) reported gambling in the last year and were included in the current study. The sample contained 696 (56.4%) men and 538 (43.6%) women. The average age was 38.5 (SD = 15.2). More than half (*n* = 677; 58.1%) were married, one-third were single (*n* = 393; 33.7%), and 96 (8.2%) were divorced or widowed. Half the sample reported low economic status (*n* = 592; 50.8%), a quarter average (*n* = 298; 25.6%), and a quarter high (*n* = 276; 23.7%) (85 participants chose not to answer these questions).

Clarification: Not all participants responded to every question. Consequently, the percentages presented correspond to the total number of respondents for each specific question.

The sociodemographic characteristics by categories of gambling severity are presented in Table 1.

**Table 1:** Socio-demographic characteristics by categories of the continuing gambling severity (*N* = 1,251)

	Non-problem gambling (*n* = 746) N (%)	Low-risk gambling *(n* = 319) N (%)	Moderate risk gambling *(n* = 141) N (%)	Problem gambling *(n* = 45) N (%)	
***Age***					
***18–28*** **(a)** ***29–41*** **(b)** ***42+*** **(c)**	**199 (51.3%) (c)** **233 (55.9%) (c)** **314 (70.4%) (a, b)**	**113 (29.1%) (c)** 111 (26.6%) **95 (21.3%) (a)**	**58 (14.9%) (c)** **56 (13.4%) (c)** **27 (6.1%) (a, b)**	18 (4.6%) 17 (4.1%) 10 (2.2%)	χ2(6) = 40.34, p < 0.001
***Gender***					
**Male (a)** **Female (b)**	**383 (55.1%) (b)** **356 (65.9%) (a)**	183 (26.3%) 132 (24.4%)	**98 (14.1%) (b)** **41 (7.6%) (a)**	**31 (4.5%) (b)** **11 (2.0%) (a)**	χ2(3) = 23.05, p < 0.001
***Martial status***					
**Single (a)** **Married (b)** **Other (c)**	**203 (51.3%) (b, c)** **426 (63.0%) (a)** **68 (70.8%) (a)**	118 (29.8%) 162 (24.0%) 21 (21.9%)	**59 (14.9%) (c)** 68 (10.1%) **5 (5.2%) (a)**	16 (4.0%) 20 (3.0%) 2 (2.1%)	χ2(6) = 21.83, p < 0.001
***Religiosity***					
**Secular** Traditional Religious Orthodox	214 (62.2%) 133 (53.0%) 314 (61.9%) 64 (57.7%)	88 (25.6%) 73 (29.1%) 123 (24.3%) 25 (22.5%)	33 (9.6%) 34 (13.5%) 53 (10.5%) 17 (15.3%)	9 (2.6%) 11 (4.4%) 17 (3.4%) 5 (4.5%)	χ2(9) = 10.32, p > 0.05
***Economic status***					
**Low (a)** **Average (b)** **High (c)**	**319 (53.7%) (b, c)** **19 (63.7%) (a)** **187 (68.2%) (a)**	**168 (28.3%) (c)** 77 (25.7%) **56 (20.4%) (a)**	81 (13.6%) 28 (9.3%) 23 (8.4%)	26 (4.4%) 4 (1.3%) 8 (2.9%)	χ2(6) = 23.95, p < 0.001

*a, b, c = bonferroni adjustment for *post hoc* tests following Chi-square test, to show exactly in which group significant differences were found

### Measures

The questionnaire included the following:

### Demographic characteristics

The participants were asked about their age, gender, marital status, religiosity, and economic status.

### The gambling behavior questionnaire - includes types, venues, and legality

The gambling behavior questionnaire was developed for the present study and included the common types of gambling in Israel [9], and it was also based on the format proposed by Tessler et al. (2017) for examining legal or illegal gambling types and behavior [27]. The gambling behavior main scale consisted of the 2 major types of legal gambling in Israel: sports bets - physical or online; and lottery or lucky cards -physical as operated by the official operator in Israel by ‘Mifal Hapais’. However, there is also a way to participate in legal lottery online by being a member of a ‘club lottery group’ without the permission of the official operator.

The gambling behavior main scale also consisted of 6 other types of illegal gambling in Israel: sports bet, casino, poker, animal betting, dice, bingo, and “other option” (to cover any other form of gambling activity not included in the other combinations that the participant wanted to add). In each type of legal or illegal gambling, the participants were asked if they gambled during the last year, and the answers were yes or no. Those who had participated at least once in each activity were asked about the venues where those gambling behaviors occur - online or physical on-site (Israel, abroad, or on a cruise).

### Problem gambling severity index (PGSI)

In order to examine Israel’s gamblers’ gambling behavior on the continuum of severity we incorporated the problem gambling severity index (PGSI) [34] into the questionnaire for this study. The PGSI consists of nine questions assessing the extent of gambling-related harm experienced over the previous 12 months. Respondents answered options on a 4-point scale: ‘never’ (0), ‘sometimes’ (1), ‘most of the time’ (2), and ‘almost always’ (3). The PGSI is scored between zero and 27. The scoring of gamblers is classified into four gambling categories: a total score of zero indicated ‘Non-problem gambling’; a score of 1–2 ‘Low-risk gambling’; a score of 3–7 ‘Moderate-risk gambling’ and a score of 8+ ‘Problem gambling’. Cronbach’s α = 0.826.

### Attitudes toward legalization of casinos and poker in Israel

Two questions have been developed for the questionnaire to assess attitudes both in favor of and against the legalization of casinos and poker in Israel. The answer scale for each question ranges from 1 - “very much in favor” to 4 - “very much against”.

### Cannabis and other illegal substance use

Two questions examined cannabis and other illegal substance use, all coded as yes or no: (1) cannabis use ever and (2) other illegal substance use ever.

## Procedure

The questionnaires were delivered online via Maskar Institute (which specializes in conducting surveys in Internet panels) to approximately 15,000 Jewish-Israeli adults belonging to a representative stratified sample according to age and gender. A total of 3,088 participants answered the questionnaire. Of these, 1,251 reported gambling activity in the last year and were included in the analyses.

The Ethical Committee of the Institutional Review Board (IRB) from the author’s institute approved the research study (AU-SOC-HBN-20191225-2). All the participants were informed about the study and confirmed their informed consent. The online questionnaire was distributed in June 2020, approximately three months after the beginning of the outbreak of COVID-19. The participants confirmed their informed consent to conduct the study. Participants were told that one product coupon will be raffled off among them. They were guaranteed anonymity, clarifying that they could stop answering questions at any time.

### Data analysis

Analyses were carried out using SPSS Version 25. The participants were divided into four groups according to the problem gambling severity index (PGSI): ‘non-problem gambling’, ‘low-risk gamblers’, ‘moderate risk gamblers’, and ‘problem gambling’. Socio-demographic differences between these four groups were assessed through Chi-square testing followed by Bonferroni adjustment. Chi-square analyses were conducted to assess differences between groups due to venues, legality, attitudes, and substance use. Logistic regression was conducted to identify medium-risk and problem gambling based on sociodemographic variables, venues, and legality.

## Results

The most common type of gambling among Israeli gamblers is the legal National Lottery or Lucky Cards. This is the official physical Israeli lottery, with 84% reporting participation in the last year and 11% in legal but unsupervised online lottery - that is, not operated by the official lottery. Of the participants, 22% reported participating in legal sports betting (physical or online), and 1.3% reported participating in illegal sports betting.

Of the participants, 10% reported gambling at legal casinos abroad, 2% reported gambling at a legal casino on a cruise, and 0.7% reported participating in illegal casinos in Israel. Of the participants, 10% reported participating in illegal poker, and 2% reported participating in legal poker abroad. Of the participants, 1.2% reported participating in illegal animal betting, and 0.3% reported participating in legal animal betting abroad. Of the participants, 2.5% reported participating in illegal dice gambling, 0.7% gambled legal dice abroad, and 0.8% reported participating in illegal bingo.

### Gambling behavior on the continuum of severity

Of the 1,251 people who reported participating in any type of gambling in the last year, 60% (*n* = 746) were classified as ‘non-problem gamblers’, 25% (*n* = 319) were classified as ‘low-risk gamblers’, 11% (*n* = 141) were classified as ‘moderate risk gamblers’, and 4% (*n* = 45) were in the ‘problem gambling’ category.

According to the all-representative sample of 3,088 Jewish-Israeli adults who answered the questionnaire (including gamblers and nongamblers), the rate of people with ‘moderate risk gambling’ was 5%, and that of ‘problem gambling’ was 1.5%.

### Problem gambling severity by venues

Participants were divided into two groups according to the venues of gambling: online or physical. Among the participants, 17% (*n* = 210) reported gambling in online venues.

**Fig. 1:**
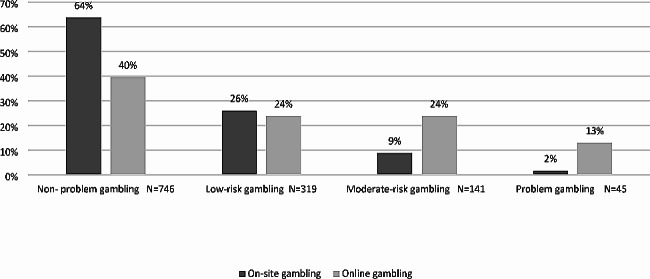
Problem Gambling Severity in the last year by venues (*N* = 1,251)

The problem of gambling severity by venues in the last year is presented in Fig. 1.

As presented in Fig. 1, there is a significant but weak association (Cramer’s v = 0.30) that was found between gambling behavior on the continuum of severity and venues of gambling: online or physical (χ2(3) = 114.0, *p* < 0.001).

The category that does the most online gambling is the ‘non-problem gambling’ group (40%; *n* = 83). However, significant differences were found in this category between online gamblers, as most of the sample reported participants in physical gambling (64%; *n* = 658). A quarter (24%; *n* = 50) of the online gamblers were found in ‘low-risk gamblers’ as well as in the ‘moderate risk gamblers’ category. However, significant differences were found only in the ‘moderate risk gamblers’ category between online gamblers and physical gamblers (9%; n = 91). Only 13% were participants in online gambling among people who were diagnosed with ‘problem gambling’, as significant differences were found between them and physical gamblers (2%; n = 17).

### Problem gambling severity by legality

Participants were categorized as illegal gamblers if they had gambled at least once in any illegal gambling type in the last year and legal gamblers if they gambled in any type of legal gambling but never in illegal gambling. Among the participants, 13% (*n* = 161) reported illegal gambling.

**Fig. 2:**
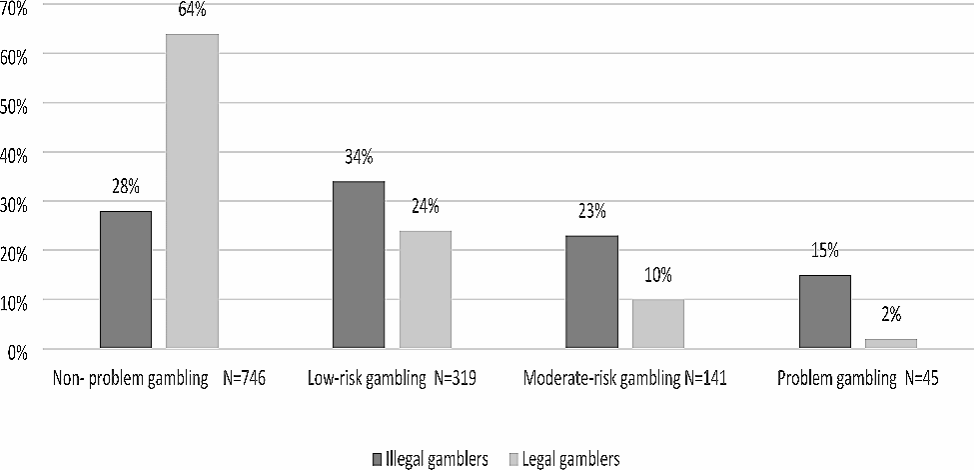
Problem gambling severity in the last year by legality (*N* = 1,251)

The problem gambling severity in the last year by legality is presented in Fig. 2.

As presented in Fig. 2, there is a significant but weak association (Cramer’s v = 0.32) that was found between gambling behavior on the continuum of severity (four groups) and legality: illegal and legal gambling (χ2(3) = 126.0, *p* < 0.001).

The group that participated most in illegal gambling was ‘low-risk gamblers’ (34%; *n* = 54), but no significant differences were found between illegal and legal gamblers in this category (24%; *n* = 265). After, found the category of ‘non-problem gambling’ (28%; *n* = 45), with significant differences found between illegal and legal gamblers in the same category (64%; *n* = 701). Almost a quarter (23%; *n* = 37) of the illegal gamblers were found in the ‘moderate risk gamblers’ group, and significant differences were found between them and legal gamblers (10%; n = 104) in this category. Only 15% were illegal gamblers among people who were diagnosed with ‘problem gambling’, but significant differences between them and legal gamblers (2%; n = 21).

### Problem gambling severity by substance use

**Fig. 3:**
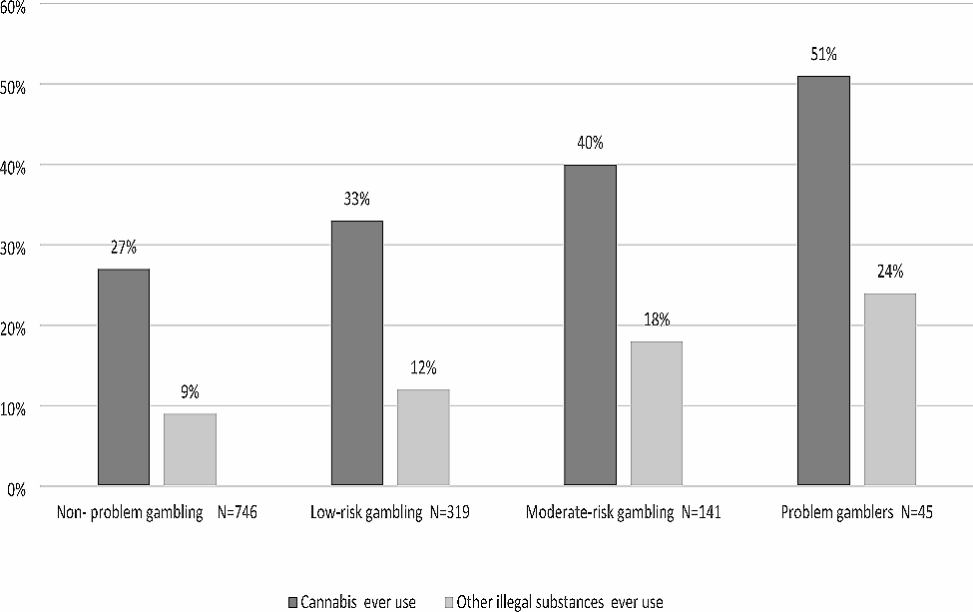
Problem Gambling Severity in the last year by substance use (*N* = 1,251)

Among the participants, 30.5% (*n* = 382) reported ever using cannabis, and 11% (*n* = 139) reported ever using other illegal substances. The problem gambling severity in the last year by cannabis and other illegal substance use is presented in Fig. 3.

Significant but weak associations (Cramer’s v = 0.13) were found between those four groups, as gambling behavior was more severe and was significantly associated with ever using cannabis (χ2(3) = 21.0, *p* < 0.01) and other illegal substances (χ2(3) = 20.19, *p* < 0.01).

The group that participated most in cannabis use (51%; *n* = 23) and other illegal substance use (24%; *n* = 11) was those diagnosed with ‘problem gambling’. People with ‘non-problem gambling’ were the lowest in cannabis use (27%; *n* = 198) and other illegal substance use (9%; *n* = 65).

### Attitudes toward legalization of casinos and poker in Israel

Among the participants, 33% (*n* = 410) reported ‘very much in favor’ and ‘in favor’ (positive) attitudes toward legalized casinos, and 32% (*n* = 402) reported ‘very much in favor’ and ‘in favor’ (positive) attitudes toward legalized poker in Israel. Additionally, a third reported ‘very much against’ and ‘against’, and a third reported ‘Neither for nor against – neutral’.

A significant but weak association (Cramer’s v = 0.13) was found between gambling behavior on the continuum of severity, positive attitudes and support for the legalization of casinos (χ2(3) = 20.91, *p* < 0.01) in Israel. A significant but weak association (Cramer’s v = 0.19) was found between gambling behavior on the continuum of severity, positive attitudes and support for legalizing poker (χ2(3) = 42.27, *p* < 0.01) in Israel.

Half of the people (50%; *N* = 22) who were classified with problem gambling with high gambling severity (8+) supported the establishment of a legal casino in Israel, and 64% of them (*N* = 28) supported the legalization of poker in Israel. More than a third of people who were ‘moderate risk gamblers’ and ‘low-risk gamblers’ supported the establishment of a legal casino (38% & 40% accordantly) and legalized poker (39%), as only 28% (n = 209) of people with ‘non-problem gambling’ supported the establishment of a legal casino and 26% (n = 194) supported the legalization of poker.

Logistic regressions to predict ‘medium risk’ and ‘problem gambling’ are presented in Table 2.

**Table 2:** Logistic regressions to identify medium risk and problem gambling (*N* = 1,251)

Variable	Value	**OR**	**95% C.I.for OR**		P
			Lower	Upper	
Gender	Men- 1, Women- 0	**2.2**	1.5	3.3	0.000
Age group	29–41–1, 42+-0	**2.8**	1.6	5.0	0.000
	18–28–1, 42+-0	**2.6**	1.6	4.2	0.000
Family Status	Married-1, Single-0	0.9	0.6	1.4	0.710
	Widower/Divorced-1, Single-0	0.6	0.2	1.6	0.305
Religious status	Secular-1, Orthodox-0	0.8	0.4	1.5	0.508
	Traditional-1, Orthodox-0	1.3	0.7	2.4	0.444
	Religious-1, Orthodox − 0	0.8	0.5	1.5	0.552
Income level	Below average − 1, average − 0	1.5	0.9	2.4	0.084
	Above average − 1, average − 0	1.3	0.7	2.2	0.378
**Nagelkerke **(*R*^2^)**= 0.092**					
**Venues & Legality**					
Venues	Online − 1, terrestrial − 0	**3.8**	2.6	5.4	0.000
Legality	Illegal − 1, Legal-0	**3.3**	2.2	4.9	0.000
**Nagelkerke **(*R*^2^)**= 0.15**					
**Type of legal gambling & venues**					
Legal sports bets online	Yes-1, no-0	**2.77**	1.56	4.91	0.000
Legal sports bets, terrestrial	Yes-1, no-0	**1.79**	1.18	2.70	0.006
Legal Lotteries online (not by the official operator)	Yes-1, no-0	**1.79**	1.10	2.90	0.019
Legal lotteries, terrestrial	Yes-1, no-0	**0.27**	0.17	0.44	0.000
**Nagelkerke **(*R*^2^)**= 0.13**					

The outcome of the logistic regressions for the association of ‘medium risk’ and ‘problem gambling’ with different study variables, including sociodemographic variables, venues, legality, and type of legal gambling.

People with ‘Medium risk’ and ‘problem gambling’ showed stronger associations with online gambling (OR = 3.8; CI 2.6–5.4; *p* < 0.000), illegal gambling (OR = 3.3; CI 2.2–4.9; *p* < 0.000), the age group of 29–41 years old (OR = 2.8; CI 1.6–5.0; *p* < 0.000), legal sports bets online (OR = 2.77; CI 1.56–4.91; *p* < 0.000), the age group of 18–28 years old (OR = 2.6; CI 1.6–4.2; p < = 0.000), and men (OR = 2.2; CI 1.5–3.3; p < = 0.000).

## Discussion

The objectives of this study were to examine gambling behavior on the continuum of severity among gamblers in Israel and to examine the association between gambling behavior on the continuum of severity to venues, legality, attitudes toward the legalization of casinos and poker and cannabis and other illegal substance use.

The findings of the current study show that most of the participants were classed with ‘non-problem gambling’ or were ‘low-risk gamblers’ (85%). Those findings strengthen findings from other studies [35] that suggest that many individuals participate in gambling as a social activity and for fun without any adverse consequences [1, 2]. Among the gambler participants, 4% identified with ‘problem gambling’. However, according to all 3,088 people who answered the questionnaire (gamblers and nongamblers) from a representative sample (due to the age and gender of the Jewish population in Israel), the rate of ‘problem gambling’ is, in fact, 1.5% of the population. This places Israel closer to the lower end of the spectrum in terms of the prevalence of problem gambling when compared to the global rate, which ranges from 0.12–5.8% [1, 11].

However, that low rate is still higher compared to Britain, where, for example, the gambling market is legal, with a problem gambling rate of only 0.2% [36]. This can also be explained by the fact that Israel is considered a more conservative country in the context of gambling and only certain types of gambling are permitted, and little attention is given to gambling behaviors by policymakers and researchers [9, 13]. Perhaps prohibited gambling behaviors are more attractive, as it was also found in the current study that 15% of the illegal gamblers were diagnosed with ‘problem gambling’ and significant differences were found between them and legal gamblers (2%) in this category. This explanation is also strengthened by comparing the category of the ‘moderate risk gamblers’, as Israel identified 5% from the representative population sample, while in Britain there is only 1.3% of this gambler type [36]. Additionally, consistent with other studies, most ‘problem gambling’ was higher among men than women [28, 29].

### Problem gambling severity by legality

The findings of the current study show that most of the people who gamble did so in legal gambling, as only 13% reported gambling in illegal gambling. This is consistent with other studies that show that most of the population prefers to be involved in legal rather than illegal behaviors [15].

In the current study, the category that used the most illegal gambling was ‘low-risk gamblers’ (34%), followed by ‘non-problem gamblers’ (28%), ‘moderate risk gamblers’ (23%), and ‘problem gamblers’ (only 15%). These findings show that not all illegal gambling is associated with serious gambling behavior; in contrast, most illegal gamblers (62%) are at the end of the less risky continuum for gambling behavior (‘non-problem’ and ‘low-risk’ gambling). The reasons for low-risk and non-problem gamblers participating in illegal gambling may be lack of understanding and awareness of which type of gambling is considered illegal in Israel. A previous qualitative study found a great deal of confusion regarding the legal status of poker and some types of online gambling in Israel [9]. Another reason may have to do with the legal consequences. The police pay only occasional attention to Illegal gambling. It is possible that lack of enforcement and the low punishment for gambling offenses, which are considered misdemeanors, are the reason for the flourishing of illegal gambling in the general population in Israel, not only among problem gamblers [9, 13].

As recent findings also identified that other categories on the continuum of severity of gambling can harm and affect others [23, 24], the results of this study also suggest that more focus is needed on other categories of gambling behaviors. However, as problematic gambling and health risks related to gambling are higher among illegal gamblers than among legal gamblers [13, 27], in the current study, when examining the end of the more risky continuum for gambling behavior, which considers more risky behaviors from others (‘moderate risk’ and ‘problem gamblers’), those gamblers showed stronger associations with illegal gambling than with legal gambling.

### Problem gambling severity by venues

The findings of the current study show that most of the people who gamble did so in physical venues, as only 17% reported gambling online. The low rate of online gambling in Israel can be explained by the fact that most legal online betting is prohibited in Israel, as only some kinds of sport betting is permitted. In Britain, for example, where the online gambling market is legal, online gambling was 27% percent among adults over a period of the previous four weeks [36].

In the current study, the category that uses the most online gambling is ‘non-problem gambling’(40%), followed by ‘low-risk gamblers’ (24%) and ‘moderate risk gamblers’, and only 13% were participants in online gambling among people who were diagnosed with ‘problem gambling’. These findings show that not all gamblers online are associated with serious gambling behavior; in contrast, most online gamblers (64%) are at the end of the less risky continuum for gambling behavior (‘non-problem’ and ‘low-risk’ gambling). As some people who start off as low gamblers may progress along the continuum and develop difficulties with gambling [35], and findings from recent studies identified that other categories on the continuum of severity need more focus [23, 24], the results of this study also suggest the same conclusion. However, previous studies have found that online gambling is potentially more addictive than physical on-site gambling [12]. Additionally, in this study, when examining the end of the riskier continuum for gambling behavior, which considers more risky behaviors from others (‘moderate risk’ and ‘problem gambling’), gamblers showed stronger associations with online gambling than with physical on-site gambling.

### Problem gambling severity by substance use

The findings of the current study show that 30.5% reported using cannabis and 11% reported using other illegal substances. Comparing those data to the last National Epidemiological Survey published in 2017 [37], the estimate for the adult population aged 18–65 years, cannabis use in the last year (no reported use ever) was 27%, and other illegal drugs were 2%.

In another national survey conducted only on cannabis use, the estimate for the adult population aged 18 + was 24% ever use [38]. It seems that the rate of cannabis use is close to the national data, although according to other illegal substances, the rate among people who gambled in the last year is higher.

The current study found that as gambling behavior became more severe, it was significantly associated with substance use. The group that participated most and the highest cannabis (51%) and other illegal substance (24%) users were the people with ‘problem gambling’ category, as the ‘non-problem gambling’ were the lowest in cannabis (27%) and other illegal substance use (9%).These finding are consistent with studies that present a possible association between gambling disorder and addiction-related substance abuse [30] and high levels of substance use among people with problem gambling [32, 33].

### Attitudes toward legalization of casinos and poker in Israel

The findings of the current study show that one-third of the participants reported positive attitudes toward legalized casinos and poker in Israel. As gambling behavior became more severe, it was significantly associated with a positive attitude toward legalizing casinos and poker in Israel. The group that most supported casinos (50%) and poker (64%) was the people with ‘problem gambling’ category, as the ‘non-problem gambling’ were the lowest in legalized casino (28%) and poker (26%) support.

As, in the current study, 10% reported gambling at legal casinos abroad and 2% reported gambling at a casino on a legal cruise, and despite the regulations concerning gambling, illegal gambling is flourishing in Israel, Israel should establish a comprehensive gambling policy and conduct public discourse about establishing casinos and poker in Israel.

### Limitations

As this study was conducted among Jewish-Israeli adults, it does not represent other Israelis groups such as Arabs. Additionally, due to the self-selected nature of the online survey, there is the possibility of response bias, and the results should not be considered representative of all gamblers in Israel. Also, the findings may primarily be applicable to the Israeli context, and caution should be exercised when generalizing these results to different cultures. Furthermore, participants in the study were not queried about the frequency of their gambling activities or their expenditure. These aspects should be investigated in future studies. However, the study boasts several strengths. Firstly, it drew upon a substantial and diverse sample of 3,088 Israeli adults. The deliberate inclusion of 1,251 participants (40.5%) who reported gambling in the last year enhances the study’s robustness, ensuring a thorough exploration of gambling behaviors within an actively engaged segment of the population. Additionally, the study’s focus on various dimensions of gambling behavior, including severity, venues, legality, and substance use, contributes to a holistic understanding of the complex landscape of gambling within Israeli society.

## Conclusions

This study sheds light on gambling behavior in Israel. This study’s findings offer evidence-based insights to shape health policy, emphasizing the need for prevention and harm reduction interventions for Israeli gamblers. The findings emphasize the critical need to broaden our focus beyond problematic gambling, extending attention to encompass also low- and moderate-risk behaviors. Of particular concern are the realms of online and illegal gambling, which demand careful consideration in the formulation of comprehensive health policies and targeted interventions.

Lowering Israel’s gambling rate could be achieved through various policies and interventions. Implementing stricter regulations on gambling advertisements, particularly targeting vulnerable populations, could help reduce the accessibility and normalization of gambling activities. Additionally, providing comprehensive education and awareness programs about the potential risks and consequences of gambling addiction could help deter individuals from engaging in excessive gambling behaviors.

The study highlights the association between illegal gambling and low-risk gamblers, underscoring the complexity of illegal gambling behaviors within the population.

Equally significant is the uncovered link between gambling behaviors and illegal substance use. The correlation signals a convergence of risky behaviors that necessitates a holistic approach to public health interventions.

This study lays the groundwork for evidence-based policies that consider the multifaceted dimensions of gambling behavior. Furthermore, the research highlights the relevance of public attitudes toward the legalization of casinos and poker in Israel. Understanding these attitudes is crucial for developing regulations that align with societal values and preferences.

More research attention should be paid to all categories on the continuum of severity, and harm reduction interventions should be initiated.

## Data Availability

The database for this study is not publicly available. It could be available from the author on reasonable request.

## Abbreviations

DSM: Diagnostic and Statistical Manual of Mental Disorders
GA: Gamblers Anonymous;
GD: Gambling Disorder
ICD: International Classification of Diseases
ISBB: Israel Sports Betting Board
PGSI: Problem Gambling Severity Index
UNODC: United Nations Office of Drugs and Crime
WHO: World Health Organization
